# Brain Temperature in Spontaneously Hypertensive Rats during Physical Exercise in Temperate and Warm Environments

**DOI:** 10.1371/journal.pone.0155919

**Published:** 2016-05-23

**Authors:** Lucas Rios Drummond, Ana Cançado Kunstetter, Filipe Ferreira Vaz, Helton Oliveira Campos, André Gustavo Pereira de Andrade, Cândido Celso Coimbra, Antônio José Natali, Samuel Penna Wanner, Thales Nicolau Prímola-Gomes

**Affiliations:** 1 Laboratório de Biologia do Exercício, Departamento de Educação Física, Universidade Federal de Viçosa, Viçosa, MG, Brasil; 2 Laboratório de Endocrinologia e Metabolismo, Departamento de Fisiologia e Biofísica, Universidade Federal de Minas Gerais, Belo Horizonte, MG, Brasil; 3 Laboratório de Fisiologia do Exercício, Departamento de Educação Física, Universidade Federal de Minas Gerais, Belo Horizonte, MG, Brasil; 4 Laboratório de Biomecânica, Departamento dos Esportes, Universidade Federal de Minas Gerais, Belo Horizonte, MG, Brasil; Cardiovascular Pathophysiology Institute, University of Buenos Aires, ARGENTINA

## Abstract

This study aimed to evaluate brain temperature (T_brain_) changes in spontaneously hypertensive rats (SHRs) subjected to two different physical exercise protocols in temperate or warm environments. We also investigated whether hypertension affects the kinetics of exercise-induced increases in T_brain_ relative to the kinetics of abdominal temperature (T_abd_) increases. Male 16-week-old normotensive Wistar rats (NWRs) and SHRs were implanted with an abdominal temperature sensor and a guide cannula in the frontal cortex to enable the insertion of a thermistor to measure T_brain_. Next, the animals were subjected to incremental-speed (initial speed of 10 m/min; speed was increased by 1 m/min every 3 min) or constant-speed (60% of the maximum speed) treadmill running until they were fatigued in a temperate (25°C) or warm (32°C) environment. T_brain_, T_abd_ and tail skin temperature were measured every min throughout the exercise trials. During incremental and constant exercise at 25°C and 32°C, the SHR group exhibited greater increases in T_brain_ and T_abd_ relative to the NWR group. Irrespective of the environment, the heat loss threshold was attained at higher temperatures (either T_brain_ or T_abd_) in the SHRs. Moreover, the brain-abdominal temperature differential was lower at 32°C in the SHRs than in the NWRs during treadmill running. Overall, we conclude that SHRs exhibit enhanced brain hyperthermia during exercise and that hypertension influences the kinetics of the T_brain_ relative to the T_abd_ increases, particularly during exercise in a warm environment.

## Introduction

Hypertension is a multifactorial clinical condition characterized by high, sustained blood pressure, often in association with functional and/or structural changes in target organs [1, 2]. As evidenced by epidemiological studies, the incidence of heat-related illness is higher in hypertensive subjects during heat waves in the United States of America and Europe [3–5]. This observation indicates that hypertension may compromise thermoregulation and hence thermal tolerance in subjects affected by this disease.

The spontaneously hypertensive rat (SHR) has been extensively used as an animal model for studying human essential or primary hypertension [6, 7]. These animals present with slow and progressive increases in total peripheral resistance [6, 8], sympathetic hyperactivity [9] and endothelial dysfunction [10, 11]. SHRs have also been shown to exhibit a greater increase in core body temperature (T_core_) when subjected to various stressful stimuli, such as heat exposure, handling or restraint [12]. Recently, we observed a greater increase in the abdominal temperature (T_abd_) of SHRs subjected to physical exercise in a warm environment. This exaggerated hyperthermia was consequent to reduced mechanical efficiency, which means that SHRs exhibited greater oxygen consumption and, consequently, greater heat production than normotensive rats when running at the same treadmill speed [13].

Most studies involving exercising rats have recorded T_abd_ or rectal temperature as an index of T_core_ [14–16]. However, T_core_ can be measured in other sites, such as the brain [17, 18]. Brain temperature (T_brain_) has been measured previously in normotensive rats during exercise in temperate and warm environments [17, 19–21]. Similar to T_abd_ and rectal temperature [14–16], T_brain_ increases in response to physical exertion in both temperate and warm environments and can reach temperatures higher than 40°C [19–21]. Because hypertension impairs brain functioning [22], T_brain_ measurements can be particularly important in SHRs. Moreover, brain heat is mainly lost through cerebral blood flow (CBF) [23], and there is evidence that CBF is inversely correlated to systemic blood pressure levels [24]. Therefore, we hypothesized that SHRs would exhibit exaggerated exercise-induced increases in T_brain_ compared with normotensive rats.

Thus, this study aimed to evaluate changes in T_brain_ in SHRs subjected to two different exercise protocols in temperate or warm environments. In addition, because T_brain_ and T_abd_ are regulated by independent physiological mechanisms [25], we also investigated whether hypertension affects the kinetics of exercise-induced increases in T_brain_ relative to the kinetics of T_abd_ increases. To achieve this second objective, we calculated the brain cortex-abdominal temperature differentials in exercising normo- and hypertensive rats.

## Materials and Methods

### Animals

Sixteen-week-old male SHRs (n = 16) and normotensive Wistar rats (NWRs; n = 16) were housed in collective cages under 14–10 h light/dark cycles in a temperature-controlled room (24°C). Initially, four animals were housed per standard polyepropylene cage with wood shaving bedding. After surgery, the rats were housed individually. Standard rat chow (Nuvilab^®^, PR, Brazil) and tap water were available *ad libitum*. The SHR group had a lower body mass relative to the NWR group at the time of the experiments (324 ± 6 g vs. 383 ± 11 g, *p* < 0.05). Taking into account that the development of hypertension in SHRs is dependent on age rather than body mass, the groups were age-matched [26]. The experimental protocol was approved by the Ethics Committee on Animal Use of the Universidade Federal de Viçosa (Protocol 58/2012) and was conducted in accordance with the Guide for the Care and Use of Laboratory Animals (2011), National Academy of Sciences (US).

Systolic (SBP) and diastolic blood pressure (DBP) was measured at the start of the experiments to confirm hypertension in the SHRs. All of the SHRs had an SBP ≥ 150 mmHg (Table 1) and were therefore considered hypertensive [7]. Blood pressure was measured by tail-cuff plethysmography (LE5001; Panlab, Barcelona, Spain). Mean blood pressure (MBP) was calculated using the following equation: MBP = DBP + 1/3(SBP—DBP).

**Table 1:** Systolic, diastolic and mean arterial blood pressure in the NWR and SHR groups.

Groups	NWR (n = 16)	SHR (n = 16)
SBP (mmHg)	120 ± 3	196 ± 4*
DBP (mmHg)	97 ± 5	155 ± 8*
MBP (mmHg)	104 ± 4	164 ± 6*

The data are expressed as the mean ± S.E.M. SBP, systolic blood pressure; DBP, diastolic blood pressure; and MBP, mean blood pressure.

* *p* < 0.05 compared with the NWR group.

### Surgical procedures

All surgical procedures were performed under intraperitoneal (i.p.) ketamine and xylazine anesthesia (80 and 10.5 mg/kg body mass, respectively). The animals were fixed to a stereotaxic apparatus, an incision was made in the skin covering the skull at the midline, and a local anesthetic with adrenaline (2% xylocaine with epinephrine) was applied to the periosteum. Next, the periosteum was excised, and craniotomy was performed using a dental drill. A sterile stainless steel cannula (13 mm length, 0.8 mm OD, 21 G) was implanted in the right frontal cortex according to the coordinates described by Paxinos and Watson [27] as follows: antero-posterior (AP): 3 mm anterior to the bregma; medio-lateral (ML): 3 mm right to the midline; and dorso-ventral (DV): 1.8 mm dorsal to the skull. The cannula was firmly anchored to the skull by two supporting screws and acrylic cement. Immediately following this procedure, a temperature sensor (G2 E-Mitter, ER4000 model, Mini-Mitter, OR, USA) was implanted in the abdominal cavity through a small incision in the linea alba. After these surgeries, the rats received an intramuscular prophylactic dose of an antibiotic (48,000 IU/kg penicillin) and a subcutaneous injection of analgesic medication (1.1 mg/kg flunixin meglumine). The rats were given 5 days to recover from surgery prior to familiarization with the treadmill exercise. This recovery period was sufficiently long for the rats to recover and regain their presurgical body mass.

### Familiarization with treadmill running exercise

The rats were familiarized with running on a treadmill (Panlab, Harvard Apparatus, Spain) by light electrical stimulation (0.2 mA). After resting for 5 min on the treadmill, the rats were made to run at a constant speed of 10 m.min^-1^ at a 5° inclination for 5 min. This running speed was increased by 1 m.min^-1^ every day. From the third day, the animals were also familiarized to running with a thermocouple fixed to their tail. The aim of these preliminary exercise sessions was to teach the rats the direction to run without becoming entangled in the thermocouple wires and to reduce their exposure to electrical stimuli during the experimental trials [28].

### Experimental design

Two sets of experiments were conducted. The first set was designed to investigate the changes in T_brain_ in the SHRs in response to incremental-speed exercise sessions. After a familiarization period, each animal was subjected to two incremental exercise protocols until it was fatigued in a temperate (25°C) or warm (32°C) environment [13]. The order of the environmental conditions was randomized and balanced. All experiments were performed between 0700 and 1300 hours. An interval of at least 48 h was given between trials. The tail skin temperature (T_skin_), T_abd_ and T_brain_ were measured every min throughout the exercise trials.

The second set of experiments was designed to investigate changes in the T_brain_ of the SHRs in response to constant-speed exercise sessions. This set of experiments was similar to the first set; however, after being familiarized to running on the treadmill, the animals underwent incremental exercise at 25°C to determine their maximum running speed (S_max_). The body temperature was not measured during this trial. The S_max_ was used to determine the speed of the animals during the constant exercises, which corresponded to 60% of S_max_. Then, on alternate days, each animal was subjected to two constant exercises until it was fatigued at 25°C and 32°C.

### Incremental-speed exercises

The thermocouple was fixed to the tail of the rats with adhesive tape, and the thermistor was inserted into the brain cortex through the guide cannula. Then, the rats were subjected to incremental running on the treadmill. The initial speed was set to 10 m.min^-1^, with increments of 1 m.min^-1^ every 3 min until volitional fatigue [29]. The inclination was maintained at 5°, and the electrical stimulation was set to 0.2 mA throughout the exercise period. The exercise was performed until volitional fatigue, which was defined as the point at which the animals were no longer able to keep pace with the treadmill for 10 s [13].

### Constant-speed exercises

The experimental procedures used during the constant-speed exercise sessions were similar to those used during the incremental exercise; the only difference was the speed, which was maintained constant at 60% of the S_max_ attained during the incremental exercise. The treadmill speed was set to 14.0 ± 0.5 m.min^-1^ and 13.3 ± 0.5 m.min^-1^ (*p* = 0.390) for the NWR and SHR groups, respectively. This low to moderate exercise intensity [30, 31] was chosen based on the criteria for prescribing exercise for hypertensive humans proposed by the American College of Sports Medicine [32].

### Control of environmental temperature during exercise

During the experiments, the dry ambient temperatures in the temperate and warm conditions were maintained at 25°C and 32°C, respectively. In the temperate environment, the ambient temperature was controlled with air conditioning (Komeco, SC, Brazil). In the warm environment, two electric heaters (Britânia, PR, Brazil) were positioned at the same level, with one in front of and the other behind the treadmill belt.

### Histological analysis

After the experiments, the rats were deeply anesthetized with i.p. ketamine and xylazine (120 and 15 mg/kg body mass, respectively). Through the ascending aorta (right atrium cut), the rats were perfused with 100 mL (10 mL.min^-1^) of heparinized (10 U.mL^-1^) phosphate-buffered saline followed by 400 mL (10 mL.min^-1^) of cold 4% paraformaldehyde (Sigma-Aldrich, St. Louis, MO, USA) in PBS using a peristaltic metering pump (Milan, PR, Brazil). The brains were removed, post-fixed in paraformaldehyde for 48 h at 4°C, and transferred to a 30% sucrose solution at 4°C for 48 h. The brains were then cut on a cryostat (Leica Microsystems, Srt. Heidelberg, Germany) into 50-μm sections and stored at 4°C in a 0.9% saline solution until being mounted on glass slides. The brain slices were stained with 0.5% cresyl violet and examined under a light microscope. The positions of the thermistor tips were confirmed by comparing the sites of the brain lesions to the neural substrates described in Paxinos and Watson’s atlas (2007).

**Fig 1:**
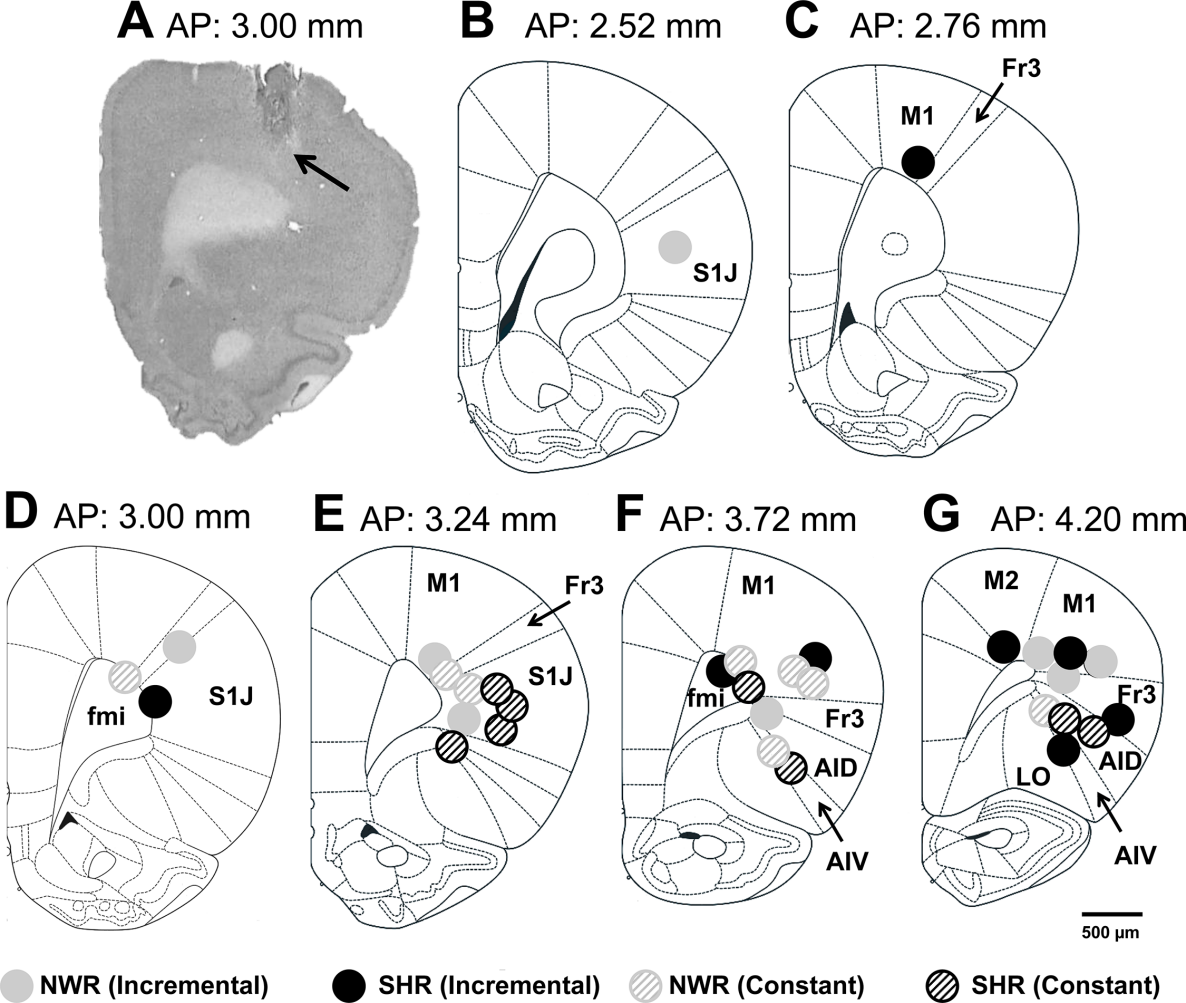
Thermistor tip positions in the brain. (A) Photomicrograph of a brain tissue section (50 μm) that was stained with cresyl violet. (B-G) Schematic drawings taken from Paxinos and Watson’s atlas (2007) showing the thermistor tip locations in the rat brain used in this study. Legend: The black arrow indicates the lesioned region. AID, agranular insular cortex, dorsal part; AIV, agranular insular cortex, ventral part; DI, dysgranular insular cortex; fmi, forceps minor of the corpus callosum; Fr3, frontal cortex, area 3; LO, lateral orbital cortex; M1, primary motor cortex; and S1J, primary somatosensory cortex, jaw region.

As shown in Fig 1 and Table 2, no differences were observed between the groups with regard to the stereotaxic coordinates at which the thermistor tips were positioned. These data indicate that the different T_brain_ responses observed in the SHRs during exercise resulted from hypertension *per se* and not from the positioning of the thermistor tips.

**Table 2:** Antero-posterior, medio-lateral, and dorsal-ventral coordinates at which the thermistor tips were positioned in the brains of SHRs and NWRs.

		Groups	
Coordinates	NWR (n = 16)	SHR (n = 16)	*p* value
Antero-posterior	3.57 ± 0.12 mm	3.70 ± 0.13 mm	0.468
Medio-lateral	2.92 ± 0.10 mm	3.00 ± 0.17 mm	0.722
Dorsal-ventral	3.49 ± 0.16 mm	3.71 ± 0.19 mm	0.401

The data are expressed as the mean ± S.E.M.

### Measurements and calculations

T_brain_ was measured using a thermistor (Beta Therm Corp., MA, USA) that was inserted through the guide cannula into the right frontal cortex. The thermistor had a diameter of 0.53 mm and was connected to equipment that measured changes in resistance (Fluke, 289 FVF, WA, USA). The resistance values (ohm) were converted into temperature values using the Steinhart-Hart equation. T_abd_ was measured by telemetry using a temperature sensor (G2 E-Mitter, ER4000 model, Mini-Mitter, OR, EUA) implanted in the abdominal cavity. This telemetric sensor sent pulses at different frequencies according to the abdominal temperature value. The radio wave frequency was captured by a receiver plate (model ER4000 energizer/receiver, Mini-Mitter) positioned next to the treadmill. T_skin_ was measured using a thermocouple fixed to the right lateral surface 1 cm from the base of the tail. The close proximity to the base of the tail enabled more sensitive measurement of changes in skin temperature that occurred as a function of changes in local blood flow (Young and Dawson, 1982). The total exercise time (TET) was measured from the start of treadmill running until volitional fatigue.

The S_max_ attained during the incremental exercise sessions was calculated by modifying the equation proposed by Kuipers et al. [33] for calculating maximal power output as follows: S_max_ = S_1_ + [S_2_(t/180)], in which S_1_ is the speed reached in the last completed stage (m.min^-1^), S_2_ is the increment in treadmill speed at each stage (m.min^-1^), and *t* is the time spent in the uncompleted stage (s). The workload was calculated using the following equation proposed by Brooks and White [34]: W = bm·g·s· senθ·TET, in which bm is the animal body mass (kg), g is the gravity force (9.8 m.s^-2^), s is the treadmill speed (m.min^-1^), senθ is the angle of treadmill inclination, and TET is the total exercise time (min). To characterize the differences in cutaneous heat loss between the experimental groups, regression equations for T_skin_ against T_core_ were calculated for each animal during exercise. The approximate location of the T_skin_ threshold was identified visually, and experimental data higher and lower than this threshold were separated. Linear regression analysis was then performed to describe the relationship between T_skin_ and T_core_, and the intersection of the regression lines (before and after) was used to determine the heat loss threshold (i.e., T_core_ at the initiation of the rapid increase in T_skin_). Heat loss sensitivity (H_sen_) was defined as the regression slope of the five time points that followed the threshold and that corresponded to the steepest part of the rising curve. Heat loss threshold (H_thr_) and H_sen_ were calculated for the two indices of T_core_ measured in this study (i.e., T_brain_ and T_abd_). The brain-abdominal temperature differentials were calculated by subtracting T_abd_ from T_brain_. This temperature differential can help to determine whether the T_brain_ changes were derived from central or peripheral sources [35, 36].

### Statistical analysis

The data are expressed as the mean ± S.E.M. The Shapiro-Wilk test was used to test all variables for normal distribution. To compare the curves for T_brain_, T_abd_ and T_skin_ between the different groups (i.e., SHR and NWR) and environment (i.e., temperate and warm) and across exercise time points, we used two-way ANOVA with a split plot, followed by the most appropriate *post hoc* test, either a t test (LSD) or a Scott-Knott test. The same statistical tests were used to compare the temperature curves between the different groups and measurement sites (i.e., T_brain_ and T_abd_) and across time points. The TET, W, H_thr_ and H_sen_ were compared between groups and environments with two-way ANOVAs followed by Tukey’s *post hoc* test. Body mass, arterial pressure and stereotaxic coordinates related to the thermistor tip positions were compared between groups using Student’s *t* tests. The effect size (ES; Cohen’s d for two dependent or independent means) was calculated by subtracting one mean from another and then dividing the result by the pooled standard deviation for the data. The significance level was set at 5%.

## Results

### Thermoregulatory responses during incremental exercise in temperate and warm environments

**Fig 2:**
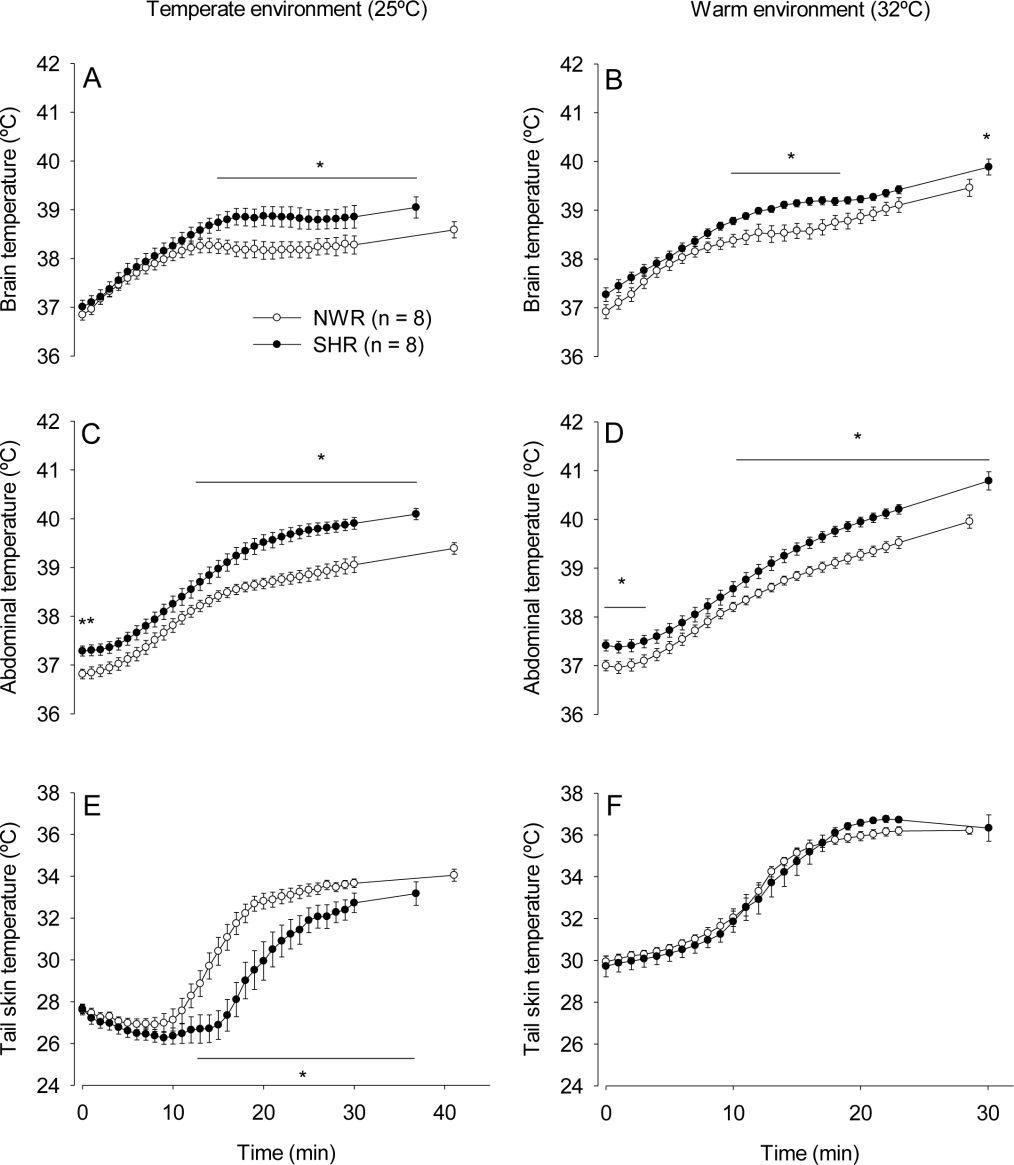
Thermoregulatory responses during incremental exercise. Brain (panels A and B), abdominal (panels C and D) and tail skin (panels D and E) temperatures of SHRs and NWRs subjected to incremental exercise in temperate (panels A, C and E) or warm (panels B, D and F) environments. Data are expressed as the mean ± S.E.M. * *p* < 0.05 compared with the NWR group in the same environment.

In response to incremental running at 25°C (Fig 2), the SHRs showed greater increases in T_brain_ relative to the NWRs from the 15^th^ min of exercise until volitional fatigue (39.04 ± 0.21°C vs. 38.58 ± 0.16°C, *p* < 0.05). For T_abd_, the SHRs showed higher temperatures than the NWRs from the onset of running (37.28 ± 0.10°C vs. 36.81 ± 0.09°C, *p* < 0.05) until the 2^nd^ min of exercise and from the 13^th^ min until volitional fatigue (40.09 ± 0.11°C vs. 39.39 ± 0.11°C, *p* < 0.05). The activation of cutaneous heat loss was markedly delayed in the SHR group, and T_skin_ increased from the 12^th^ and 17^th^ min in the NWRs and SHRs, respectively. Therefore, the SHRs exhibited lower T_skin_ from the 12^th^ min of running until volitional fatigue (28.10 ± 0.81°C vs. 31.74 ± 0.52°C at the 17^th^ min, *p* < 0.05).

In response to incremental running at 32°C (Fig 2), the SHRs exhibited a greater increases in T_brain_ than the NWRs from the 7^th^ until the 14^th^ min and at volitional fatigue (39.88 ± 0.16°C vs. 39.45 ± 0.17°C, *p* < 0.05). The SHRs showed exaggerated increases in T_abd_ compared with the NWRs from the onset (37.41 ± 0.11°C vs. 37.00 ± 0.10°C, *p* < 0.05) until the 5^th^ min of exercise and from the 10^th^ min until volitional fatigue (40.78 ± 0.18°C vs. 39.95 ± 0.13°C, *p* < 0.05). There were no differences in T_skin_ between the groups throughout the exercise at 32°C.

Ambient temperature influenced the exercise-induced increases in T_brain_, T_abd_ and T_skin_. The three body temperature values were higher at 32°C than at 25°C in both the NWR and SHR groups, including the moment when the animals became fatigued.

### Thermoregulatory responses during constant exercise in temperate and warm environments

**Fig 3:**
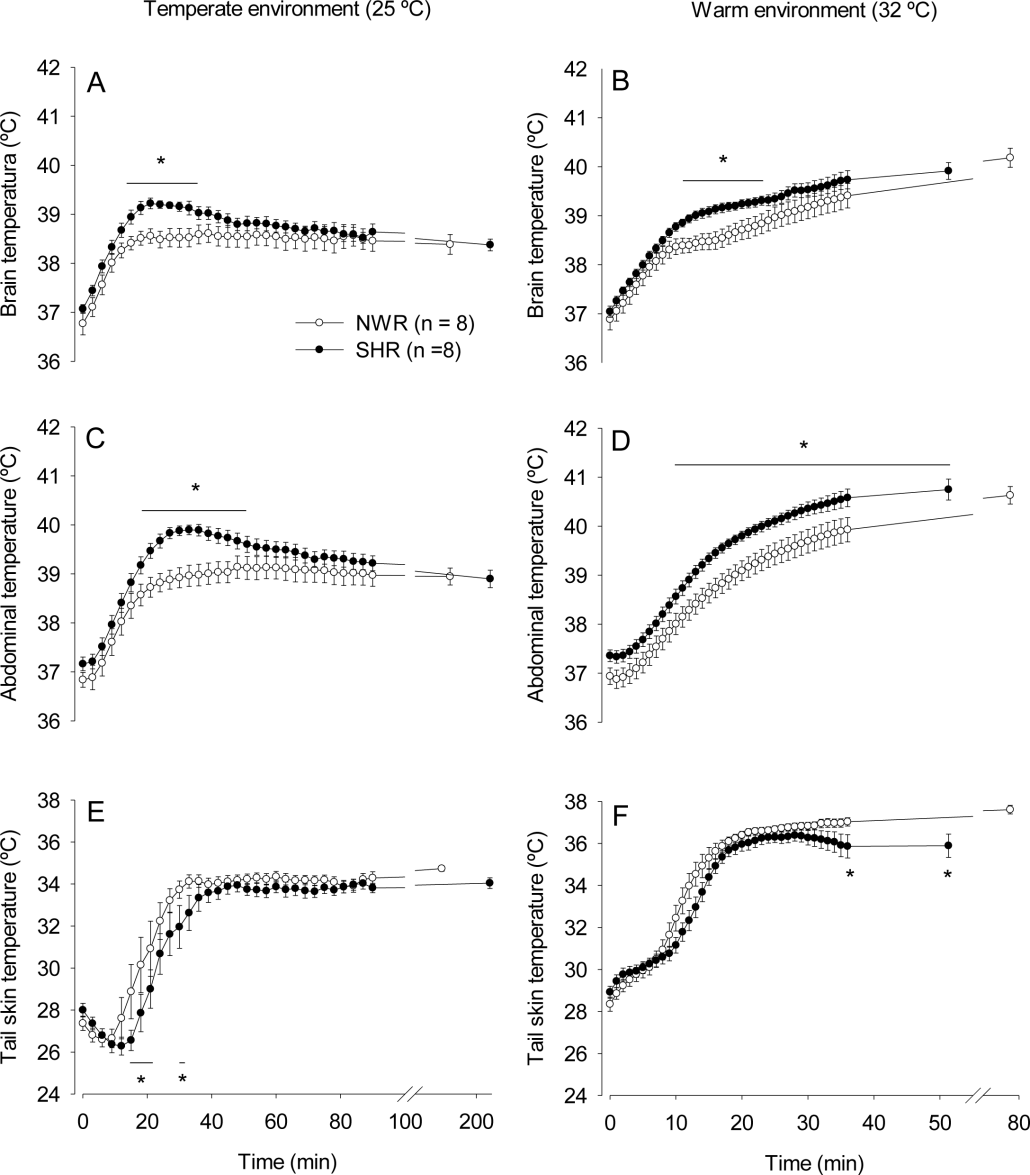
Thermoregulatory responses during constant exercise. Brain (panels A and B), abdominal (panels C and D) and tail skin (panels D and E) temperature in SHRs or NWRs subjected to constant exercise in temperate (panels A, C and E) or warm (panels B, D and F) environments. Data are expressed as the mean ± S.E.M. * *p* < 0.05 compared with the NWR group in the same environment.

In response to constant running at 25°C (Fig 3), the SHRs showed greater increases in T_brain_ relative to the NWRs from the 13^th^ until the 35^th^ min of exercise (39.06 ± 0.13°C vs. 38.57 ± 0.19°C, *p* < 0.05). For T_abd_, the SHRs exhibited higher temperatures than the NWRs from the 19^th^ until the 52^nd^ min (39.58 ± 0.16°C vs. 39.11 ± 0.26°C, *p* < 0.05). In contrast to the observations with incremental exercise trials, no differences were observed at volitional fatigue in the T_brain_ (SHR: 38.37 ± 0.11°C vs. NWR: 38.38 ± 0.20°C, *p* > 0.05) or T_abd_ (SHR: 38.89 ± 0.17°C vs. NWR: 38.94 ± 0.17°C, *p* > 0.05) of the SHRs and NWRs subjected to the constant-speed protocol. The activation of cutaneous heat loss was markedly delayed in the SHR group; T_skin_ increased from the 14^th^ and 19^th^ min in the NWRs and SHRs, respectively. Therefore, the SHRs exhibited lower T_skin_ from the 13^th^ until the 21^st^ (26.98 ± 0.62°C vs. 29.39 ± 1.35°C, at the 16^th^ min, *p* < 0.05) and from the 30^th^ until the 32^nd^ min of running.

In response to constant running at 32°C (Fig 3), the SHRs showed greater increases in T_brain_ than the NWRs from the 11^th^ min until the 24^th^ min (39.31 ± 0.11°C vs. 38.89 ± 0.21°C, *p* < 0.05). The SHRs showed greater increases in T_abd_ than the NWRs from the 9^th^ until the 36^th^ min of exercise (40.57 ± 0.18°C vs. 39.92 ± 0.24°C, *p* < 0.05). Lower T_skin_ values were observed in the SHRs compared with the NWRs at the 36^th^ min and at volitional fatigue (35.89 ± 0.55°C vs. 37.60 ± 0.20°C, *p* < 0.05).

Furthermore, ambient temperature also influenced the thermoregulatory responses induced by constant exercise. T_brain_, T_abd_ and T_skin_ were higher at 32°C than at 25°C in both experimental groups.

### Thermoeffector activity during incremental and constant exercises in temperate and warm environments

**Fig 4:**
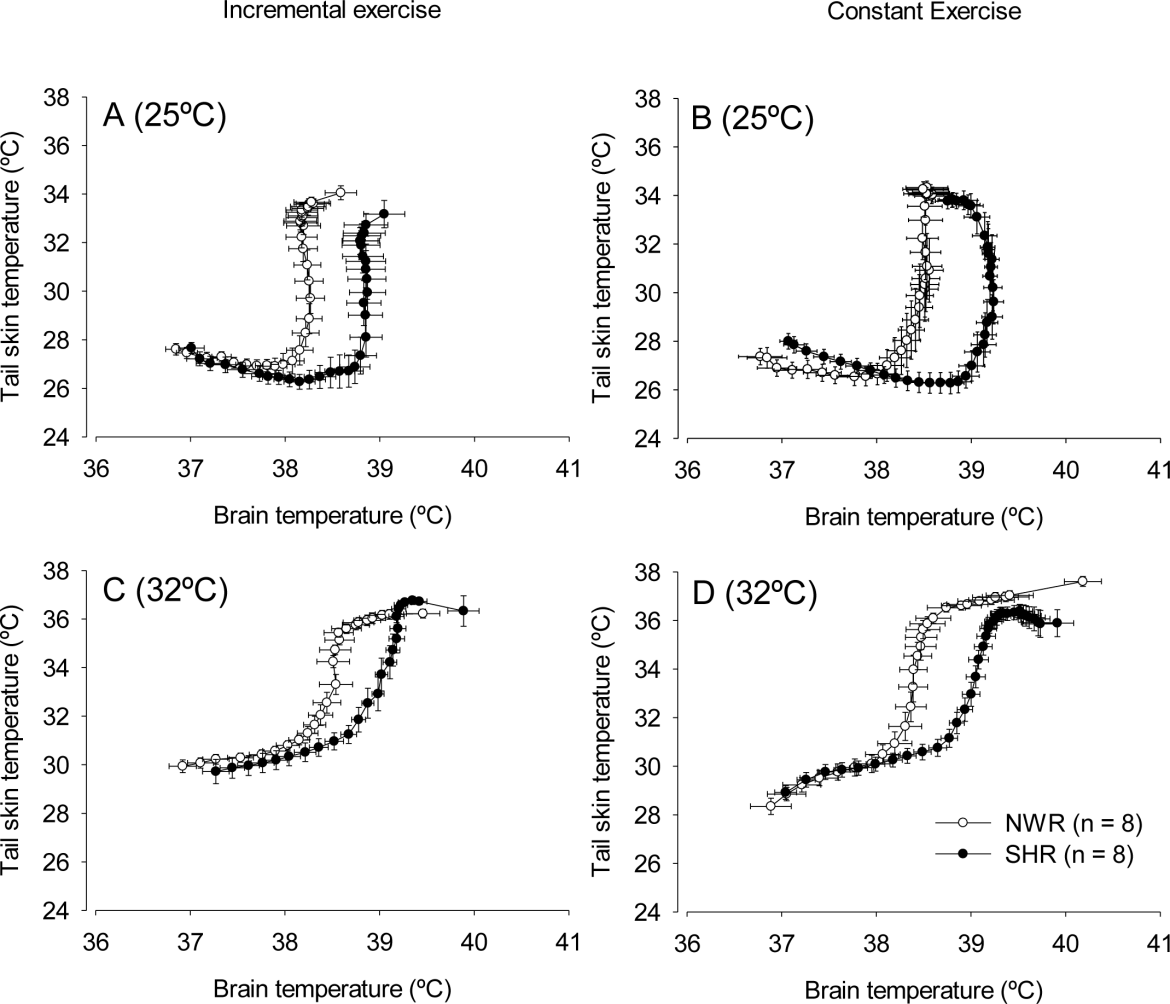
Thermoeffector activity during exercise. Thermoeffector activity during incremental (panels A and C) or constant (panels B and D) exercise in temperate (panels A and B) or warm (panels C and D) environments. Data are expressed as the mean ± S.E.M.

The curves showing the thermoeffector activity associated with cutaneous heat loss (i.e., increases in T_skin_ as a function of T_brain_ or T_abd_) were shifted to the right for the SHRs under all conditions (Fig 4). During incremental exercise at 25°C, the heat loss threshold calculated from the T_brain_ and T_abd_ data was higher in the SHRs than in the NWRs (Table 3; ES = 1.37 and 2.33, respectively). Similar findings were observed at 32°C (ES = 1.07 and 1.82, respectively, for the thresholds calculated from the T_brain_ and T_abd_ data).

**Table 3:** Heat loss threshold and sensitivity during incremental or constant exercise in temperate or warm environments.

	NWR (25°C)	SHR (25°C)	NWR (32°C)	SHR (32°C)
Incremental exercise				
T_brain_ H_thr_ (°C)	38.10 ± 0.17	38.90 ± 0.23*	38.45 ± 0.20	39.03 ± 0.17*
T_brain_ H_sen_	-3.70 ± 8.32	0.40 ± 9.00	10.66 ± 8.20	2.09 ± 6.80
T_abdominal_ H_thr_ (°C)	37.93 ± 0.12	39.03 ± 0.20*	38.03 ± 0.09	38.70 ± 0.16*
T_abdominal_ H_sen_	9.35 ± 1.58	1.58 ± 1.33	6.09 ± 0.94	5.45 ± 0.52
Constant exercise				
T_brain_ H_thr_ (°C)	38.09 ± 0.10	39.36 ± 0.13*	38.23 ± 0.17	38.98 ± 0.08*
T_brain_ H_sen_	0.21 ± 5.59	-7.66 ± 7.46	12.76 ± 5.93	-9.60 ± 13.30
T_abdominal_ H_thr_ (°C)	37.78 ± 0.15	39.16 ± 0.16*	38.03 ± 0.09	38.69 ± 0.15*
T_abdominal_ H_sen_	7.30 ± 3.97	9.47 ± 1.89	6.05 ± 0.81	5.45 ± 0.52

Data are expressed as the mean ± S.E.M. T_brain_, brain temperature; T_abd_, abdominal temperature; H_thr_, heat loss threshold; and H_sen_, heat loss sensitivity.

* *p* < 0.05 compared with the NWR group in the same environment.

During constant exercise at 25°C, the heat loss threshold calculated from the T_brain_ and T_abd_ data was higher in the SHRs than in the NWRs (ES = 3.99 and 3.16, respectively). Similar findings were observed at 32°C (ES = 1.89 and 1.72, respectively, for the thresholds calculated from the T_brain_ and T_abd_ data). Irrespective of the environment or the exercise protocol, cutaneous heat loss sensitivity did not differ between the SHR and NWR groups.

### Brain-abdominal temperature differentials during incremental and constant exercises in temperate and warm environments

**Fig 5:**
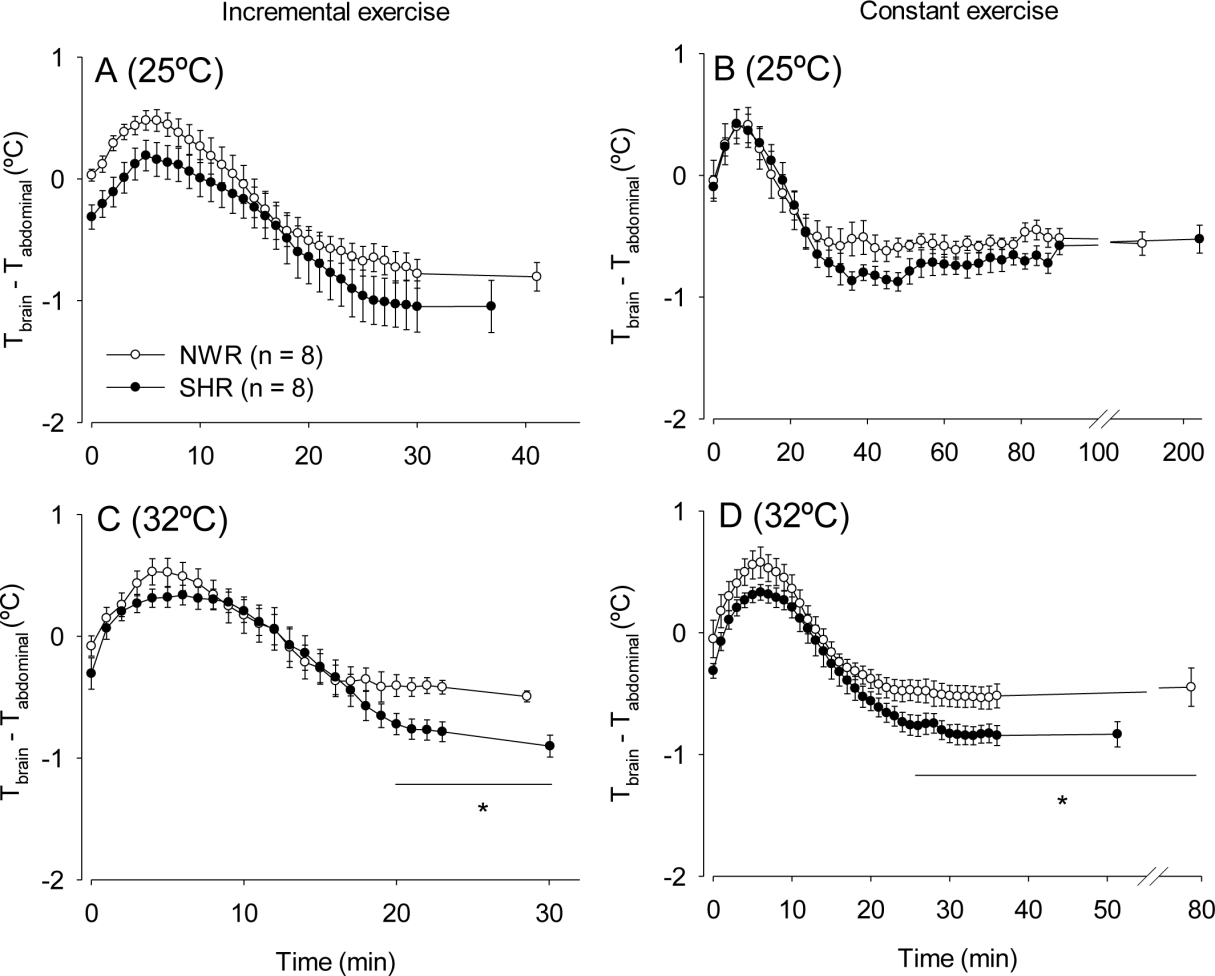
Brain-abdominal temperature differentials during exercise. Brain-abdominal temperature differentials during incremental (panels A and C) or constant (panels B and D) exercise in temperate (A and B) or warm (C and D) environments. Data are expressed as the mean ± S.E.M. * *p* < 0.05 compared with the NWR group in the same environment.

The kinetics of exercise-induced increases in T_brain_ and T_abd_ were compared between the SHRs and NWRs by calculating brain-abdominal temperature differentials (Fig 5). For example, in the NWRs subjected to incremental running at 25°C, this differential increased during the first 6 min but then decreased and became negative towards the end of the exercise period (Fig 5A). A similar pattern was observed for the increases in T_brain_ and T_abd_ in the SHRs and NWRs subjected to constant running at 25°C (Fig 5B). The kinetics of the T_brain_ and T_abd_ increases were not affected by hypertension during exercise at 25°C (Fig 5A and 5B); however, a hypertension-mediated effect was observed for either exercise protocol at 32°C (Fig 5C and 5D). The brain-abdominal temperature differential was lower in the SHRs than in the NWRs from the 20^th^ min until volitional fatigue (-0.90 ± 0.09°C vs. -0.49 ± 0.04°C, *p* < 0.05) during incremental exercise and from the 25^th^ min until volitional fatigue (-0.83 ± 0.10°C vs. -0.44 ± 0.15°C, *p* < 0.05) during constant exercise.

### Physical performances of SHR and normotensive rats

During incremental exercise, no differences were observed in the TETs between the NWR and SHR groups at 25°C or at 32°C. When physical performance was assessed by calculating workload, the SHR group demonstrated lower performance than the NWR group in the temperate (ES = 1.01) but not the warm environment (Table 4). The warm environment reduced the TET by 31% in the NWR group (ES = 2.35) and by 18% in the SHR group (ES = 1.85) relative to the TET in the temperate environment. The warm environment also reduced the workload performed by the animals in the NWR (ES = 1.77) and SHR (ES = 2.01) groups.

**Table 4:** Physical performance of the SHRs and NWRs during incremental or constant exercise.

	25°C		32°C	
	NWR	SHR	NWR	SHR
Incremental exercise				
TTE (min)	41.0 ± 3.4	36.8 ± 1.8	28.5 ± 1.6^†^	30.0 ± 1.6^†^
Workload (J)	213.0 ± 23.5	159.2 ± 12.1*	129.4 ± 8.7^†^	114.9 ± 8.3^†^
Constant exercise				
TTE (min)	177.5 ± 8.6	208.8 ± 25.3	78.8 ± 11.8^†^	51.3 ± 8.1^†^
Workload (J)	839.9 ± 48.5	665.5 ± 94.8*	377.7 ± 61.5^†^	184.3 ± 26.2*^†^

TTE, total time of exercise. Data are expressed as the mean ± S.E.M.

* *p* < 0.05 compared with the NWR group in the same environment

^†^ *p* < 0.05 compared with the same group in the temperate environment.

During constant exercise, no differences were observed in the TETs between the NWR and SHR groups at 25°C and 32°C. However, the SHR group performed less work than the NWR group in the temperate (ES = 0.91) and warm (ES = 1.44) environments (Table 4). The warm environment reduced the TET by 56% in the NWR group (ES = 6.21) and by 75% in the SHR group (ES = 3.06) and also reduced the workload in the NWR (ES = 2.49) and SHR (ES = 2.27) groups.

## Discussion

In this study, T_brain_ control was evaluated in SHRs during physical exercise in temperate and warm environments. The main finding was that SHRs subjected to treadmill running exhibited greater increases in T_brain_ during incremental and constant exercises in both environments. Additionally, SHRs subjected to exercise exhibited the following: 1) greater increases in T_abd_ during incremental and constant exercises at 25°C and 32°C; 2) higher T_core_ thresholds for triggering increases in T_skin_ during both exercise protocols at 25°C and 32°C; and 3) a lower total workload, which is a physical performance index that took differences in body mass into account, during the incremental exercise at 25°C and during the constant exercise in both environments. Furthermore, in the warm environment, SHRs exhibited distinct kinetics for T_brain_ and T_abd_ increases during the final minutes of exercise relative to those usually observed in running, normotensive rats. Taken together, these results demonstrate the existence of differences in T_brain_ control in SHRs subjected to exercise.

Our results showed that the SHRs presented greater increases in T_brain_ and T_abd_ during exercise than normotensive animals (Fig 2 and Fig 3). At 25°C, the greater exercise-induced increases in T_brain_ and T_abd_ in the SHRs can be explained, at least in part, by lower cutaneous heat dissipation to the environment (Fig 2E and Fig 3E; Table 3). In contrast with the observations at 25°C, the SHRs did not display a delayed increase in T_skin_ during exercise at 32°C (Fig 2F and Fig 3F), even though they had greater increases in T_brain_ and T_abd_. While running at 32°C, the tail skin of the rats was passively heated by the warm ambient temperature, which could have hindered the observation of inter-group differences in T_skin_ increases at the beginning of exercise. However, differences in the heat loss threshold were observed between the SHR and NWR rats at 32°C (Table 3), indicating that cutaneous heat dissipation was in fact impaired in the SHRs in the warm environment and contributed to the greater increases in T_brain_ and T_abd_ observed under these experimental conditions.

The temporal profile of thermoregulatory responses suggests an important role for the higher heat loss threshold in SHRs in the induction of their exaggerated T_brain_ and T_abd_ increases, and considering the higher metabolism of exercising SHRs [13], these differences in heat loss threshold are even more impressive. The higher threshold of the SHR group without concomitant changes in sensitivity suggests that the impaired heat loss was caused by central mechanisms. Threshold temperature shifts are often interpreted as representative of central modulation, whereas sensitivity shifts (without parallel changes in the onset threshold) imply peripheral modulation [37]. Besides these central dysfunctions that impair cutaneous tail heat loss, SHRs also present dysfunctions that impair brain heat loss. Fuji et al. [38] reported significant negative correlations between the mean arterial pressure and CBF measured in the brain cortex and the thalamus, indicating that CBF decreases with the characteristic rise in the blood pressure of hypertensive patients. Similar findings were observed in animal models developed to study hypertension. In untreated SHRs, local CBF was decreased in the brain cortex and thalamus compared with normotensive rats. In addition, long-term (18 weeks) pharmacological treatment of hypertension restored the reduced CBF to the levels measured in normotensive rats [24]. Of note, CBF is the major mechanism by which heat is removed from the brain [23], suggesting that reduced CBF in SHRs might also contribute to the greater increase in T_brain_ during exercise in these rats.

Constant exercise at submaximal intensity appeared to facilitate heat dissipation in the SHRs, which successfully, albeit with some delay, increased their T_skin_ (Fig 3A, 3C and 3E). Following the activation of cutaneous heat loss, T_brain_ and T_abd_ gradually became similar in both groups. The intensity of constant exercise corresponded to 60% of S_max_ and was selected based on the latest American College of Sports Medicine recommendations, which states that hypertensive individuals should perform exercise of low to moderate intensity [32]. Nevertheless, even when exercising in conditions of compensable heat stress (i.e., in a temperate environment and at an exercise intensity considered safe for hypertensive individuals), the SHRs had greater transient increases in T_brain_ and T_abd_ than the normotensive rats. In contrast, during the incremental exercise at 25°C, both temperature indices remained higher in the SHRs until they were fatigued (Fig 2A, 2C and 2E). The incremental exercise protocol was characterized by increases in treadmill speed that occurred in short periods of time (every 3 min) so that the animals quickly reached high running speeds. High exercise intensities led to high rates of metabolic heat production that may have exceeded the capability of the running rats to dissipate heat. Therefore, the SHRs could not adjust their T_core_ before exerting more intense efforts during incremental exercise (which is likely a condition of uncompensable heat stress), and consequently, their T_brain_ and T_abd_ remained higher than those of NWRs until volitional fatigue.

The present results indicate the existence of temporal differences in the regulation of T_brain_ and T_abd_ during exercise. Regardless of the exercise protocol, environmental temperature and strain of the rats, T_brain_ was similar to T_abd_ before exercise initiation, and the brain-abdominal temperature differential increased rapidly after the start of treadmill running but decreased within 15–20 min of exercise (Fig 5). More rapid increases in T_brain_ than in blood temperature have been reported under several stress conditions, suggesting rapid increases in brain metabolism and consequently in intra-brain heat production [25]. This hypothesis also explains the increases in the brain-abdominal temperature differential at the beginning of treadmill running (Fig 5) (14). Furthermore, exercise-mediated sympathetic outflow to the splanchnic vascular bed, which reduces blood flow in the liver, gastrointestinal tract, pancreas and spleen, could slow abdominal hyperthermia and contribute to the faster increase in T_brain_ relative to T_abd_ during the first minutes of exercise. A previous report indicated that increased blood flow in the skin and active muscles is dependent on the reduction (percentage relative to cardiac output) of splanchnic and renal blood flow [39].

The attainment of a lower T_brain_ in relation to T_abd_ during exercise suggests the existence of a mechanism that prevents large increases in T_brain_ in running rats. In this species, the most accepted theory of selective brain cooling is that cool blood draining from the nose and head skin cools the brain tissue under conditions of hyperthermia [40, 41]. Because of the increased vulnerability of the brain to increases in temperature (i.e., T_brain_ of above 40°C is associated with tissue damage and increased incidence of death) [42], the main function of selective brain cooling is to provide brain protection against thermal damage. Damage to brain cells has been documented *in vivo* during extreme environmental heating [43, 44] as a result of brain hyperthermia associated with increased permeability of the blood brain barrier and consequent vasogenic edema [25]. Nevertheless, the existence of selective brain cooling in exercising rodents is still controversial [41, 45]. Because an augmented cerebral metabolic rate might explain the more rapid increase in T_brain_ in response to arousing stimuli [25] and to exercise initiation, reduced activation of brain structures is an alternative explanation for the lower T_brain_ relative to T_abd_ after minutes of treadmill running. Thus, we cannot rule out that the differences between T_brain_ and T_abd_ were in fact consequences of reduced metabolic rates in the brain cortex when the physical effort became fatiguing. The hypertensive rats showed a lower brain-abdominal temperature differential than the normotensive rats at 32°C (Fig 5), which was likely a consequence of greater increases in T_abd_ relative to the increases in T_brain_. This observation suggests the existence of a protective mechanism to avoid exaggerated increases in T_brain_ in hypertensive rats in warm environments.

Because the body weight of the SHRs was lower than that of the NWRs, the workload was calculated to properly assess the physical performance of the rats used in this study. The workload performed by the SHRs was lower for both exercise protocols at 25°C and for constant exercise at 32°C (Table 4). The mechanisms by which hypertension reduces physical performance remain to be determined. A tentative hypothesis is that exaggerated hyperthermia may limit the exercise capacity of hypertensive rats; however, this hypothesis cannot explain the lower performance of the SHRs in all experimental trials (e.g., T_brain_ did not differ at volitional fatigue during constant exercise at 25°C; Fig 3A). Moreover, as previously suggested by Kunstetter et al. (2014), incremental exercise in a temperate environment does not appear to be a good strategy for investigating the influence of T_brain_ on performance, and under these conditions, the performance of hypertensive rats is lower than that of normotensive rats. The incremental protocol consisted of progressive and continuous increases in treadmill speed such that the animals were running close to their maximal aerobic capacity during the final moments of exercise. In this case, the ability of the cardiovascular system to specifically increase coronary and skeletal muscle blood flow is likely more of a determinant of physical performance than increases in core temperature [46].

**Fig 6:**
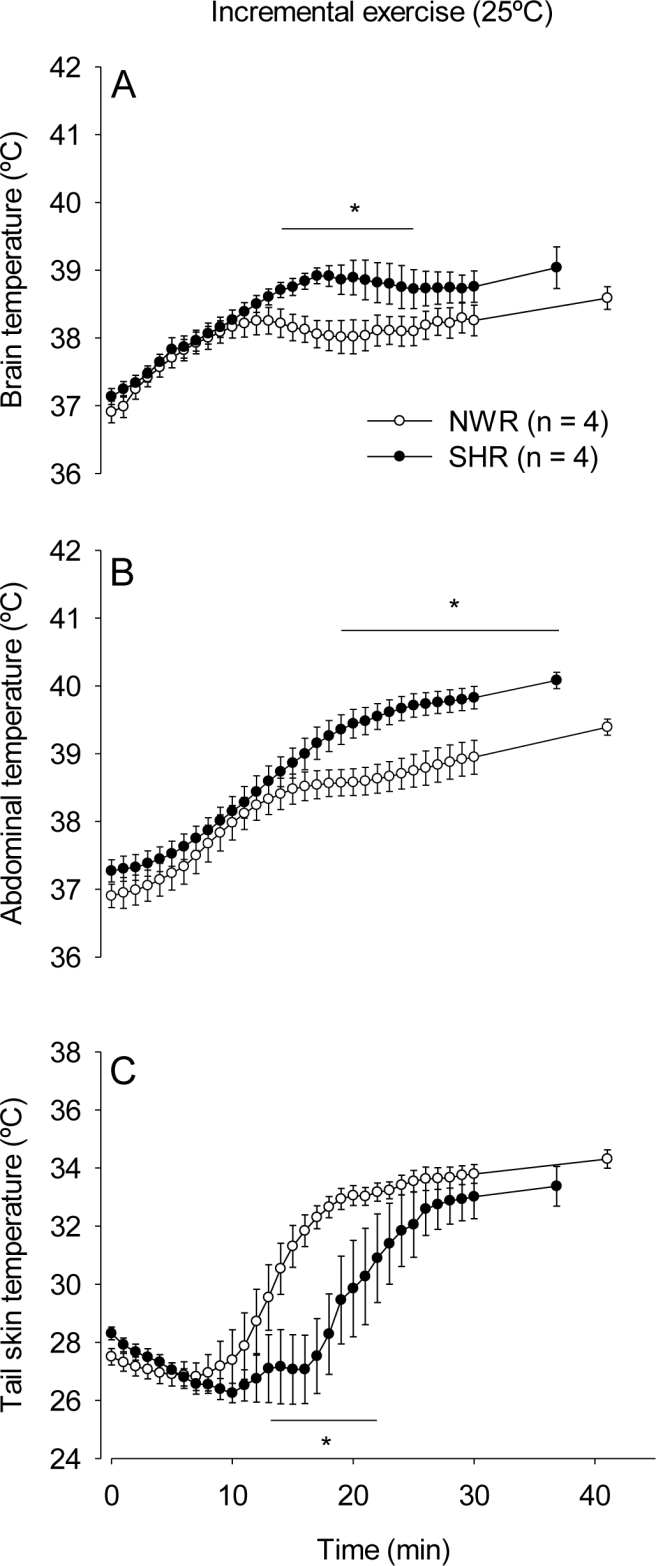
Exercise-induced thermoregulatory responses of rats from the two groups matched for body mass. (Body mass: NWR, 334 ± 10 g; and SHR, 346 ± 14 g; *p* = 0.528). Brain (panel A), abdominal (panel B) and tail skin (panel C) temperatures of the SHRs and NWRs subjected to incremental exercise in a temperate environment. Data are expressed as the mean ± S.E.M. * *p* < 0.05 compared with the NWR rats.

The difference in body mass observed between our groups could have affected the changes in T_core_ induced by exercise [47] because both the energy cost of running and cutaneous heat loss (body surface area-to-mass ratio) are dependent on body mass. To eliminate this confounding factor, care was taken to match the body masses of the two groups (the four heaviest SHRs were compared with the four lightest NWRs), and the temperature analyses were performed again. These analyses revealed that even in absence of differences in body mass, the exercise-induced increases in T_brain_, T_abd_ and T_skin_ still differed between the groups (Fig 6), suggesting that body mass was not a determinant of the inter-group differences in thermoregulation.

In conclusion, the present results indicate that the SHRs exhibited exaggerated increases in T_brain_ during both exercise protocols in temperate and warm environments. This enhanced hyperthermia can be partially explained by the higher heat loss threshold observed in the hypertensive rats during the two exercise protocols, irrespective of the environmental temperature. Furthermore, the present results reinforce the observation that T_brain_ and T_abd_ are regulated by different mechanisms in normotensive rats during exercise. However, hypertension influences the kinetics of the increase in T_brain_ relative to the T_abd_ increase during both exercise protocols in a warm environment.
